# Targeting the Janus Kinase Family in Autoimmune Skin Diseases

**DOI:** 10.3389/fimmu.2019.02342

**Published:** 2019-10-09

**Authors:** Michael D. Howell, Fiona I. Kuo, Paul A. Smith

**Affiliations:** Incyte Corporation, Wilmington, DE, United States

**Keywords:** Janus kinase, cytokines, skin barrier, dermatology, autoimmunity

## Abstract

Autoimmune skin diseases are characterized by significant local and systemic inflammation that is largely mediated by the Janus kinase (JAK)–signal transducer and activator of transcription (STAT) pathway. Advanced understanding of this pathway has led to the development of targeted inhibitors of Janus kinases (JAKinibs). As a class, JAK inhibitors effectively treat a multitude of hematologic and inflammatory diseases. Growing evidence suggests that JAK inhibitors are efficacious in atopic dermatitis, alopecia areata, psoriasis, and vitiligo. Additional evidence suggests that JAK inhibition might be broadly useful in dermatology, with early reports of efficacy in several other conditions. JAK inhibitors can be administered orally or used topically and represent a promising new class of medications. Here we review the evolving data on the role of the JAK-STAT pathway in inflammatory dermatoses and the potential therapeutic benefit of JAK-STAT antagonism.

## JAK-STAT Signaling Pathway

**Figure 1:**
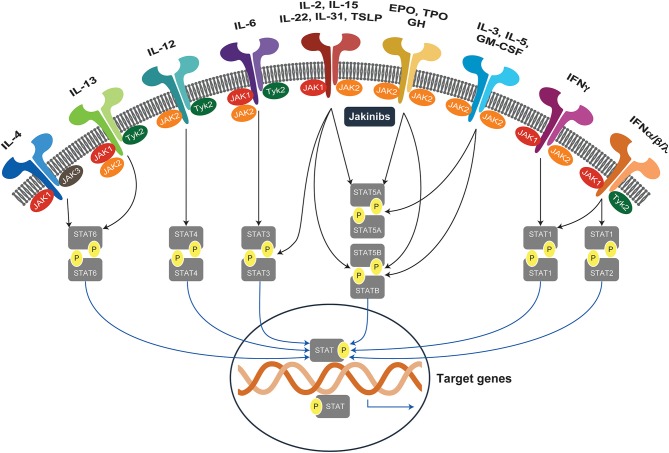
JAK-STAT signaling pathways. Janus kinases (JAK1-3, TYK2) are activated by more than 60 extracellular stimuli and phosphorylate downstream STAT proteins, which translocate to the nucleus and activate target genes. EPO, erythropoietin; GH, growth hormone; GM-CSF, granulocyte-macrophage colony-stimulating factor; IFN, interferon; IL, interleukin; JAK, Janus kinases; JAKinibs, Janus kinase inhibitors; STAT, signal transducer and activator of transcription; TPO, thrombopoietin; TSLP, thymic stromal lymphopoietin; TYK2, tyrosine kinase.

The mammalian Janus kinase (JAK) family contains three JAKs (JAK1–3) and tyrosine kinase 2 (TYK2), which selectively bind different receptor chains (1). Upon binding of ligand to its cognate cytokine receptor, associated JAKs become activated and undergo autophosphorylation and transphosphorylate the intracellular tail of their receptors. This creates docking sites for the SH2 domain of the cytoplasmic transcription factors termed signal transducers and activators of transcription (STATs). The human STAT family contains seven STATs: STAT1, STAT2, STAT3, STAT4, STAT5A, STAT5B, and STAT6. Following phosphorylation, STATs are translocated to the nucleus, dimerize, and bind to specific DNA sequences to regulate gene transcription (2). The JAK-STAT pathway is pivotal for the downstream signaling of inflammatory cytokines, including interleukins (ILs), interferons (IFNs), and multiple growth factors (3, 4). Overall, the selective use of JAKs by different receptors coupled to downstream STAT signal transduction results in an elegant mechanism to achieve exquisite *in vivo* specificity for more than 60 cytokines and growth factors (Figure 1).

Identification of selective pharmacologic JAK inhibitors (JAKinibs) has been an ongoing research and development goal. The first JAKinib to gain FDA approval in 2011 was ruxolitinib for intermediate or high-risk myelofibrosis, thereby showing that JAK inhibition was not only possible, but safe and effective for its intended uses. More recently, selective JAK inhibitors have been explored for specific inflammatory disease indications (Table 1).

**Table 1:** Selectivity profiles of clinically active JAKinibs.

**Inhibitor**	**JAK1**	**JAK2**	**JAK3**	**TYK2**
Tofacitinib^*^	X	X	X	
Ruxolitinib,^†^ Baricitinib,^‡^ Momelotinib, CTP-543	X	X		
Oclacitinib,^§^ Itacitinib, Upadacitinib, Filgotinib, PF04965842, LP0184	X			
ATI-502	X		X	
PF-06700841	X			X
PF-06651600			X	
PF-06826647 BMS-986165				X
Delgocitinib (JTE-052)	X	X	X	X

*JAK, Janus kinase; JAKinibs, inhibitors of Janus kinase; FDA, US Food and Drug Administration; TYK, tyrosine kinase*.

* *FDA approved: rheumatoid arthritis, psoriatic arthritis and ulcerative colitis*.

† *FDA approved: adults with polycythemia who have had an inadequate response to or are intolerant of hydroxyurea, adults with intermediate or high-risk myelofibrosis, acute graft-vs.-host disease in adult and pediatric patients 12 years and younger*.

‡ *FDA approved: rheumatoid arthritis*.

§ *FDA approved: atopic dermatitis and pruritus from allergic dermatitis in dogs*.

## The JAK-STAT Pathway and T Helper Subsets

**Figure 2:**
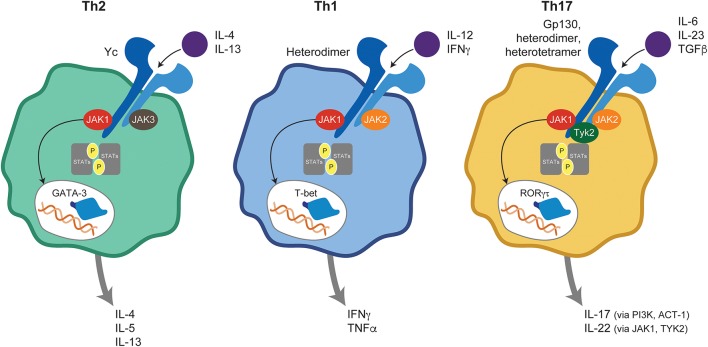
JAK-mediated cytokine signaling in T helper subsets. Ligand binding to its cognate receptor triggers JAK-STAT activation and plays a central role in naive T-cell differentiation into Th1, Th2, and Th17 subsets. ACT, Nuclear factor NF-kappa-B activator 1; GATA, GATA transcription factor 3; IFN, interferon; IL, interleukin; JAK, Janus kinase; PI3K, Phosphoinositide 3-kinases; RORγt, retinoic acid receptor-related orphan receptor γ; STAT, signal transducer and activator of transcription; T-bet, T-box transcription factor TBX21; Th, T helper; TGF, transforming growth factor; TNF, tumor necrosis factor; TYK, tyrosine kinase.

The differential fate of naive T cells into committed T helper (Th) subsets is orchestrated under the instruction of professional antigen-presenting cells within a JAK-STAT–dependent cytokine milieu (Figure 2). *In vivo* Th1 differentiation depends on JAK-mediated signaling through the IFNγ receptor (IFNGR), the IL-12 receptor (IL-12R), and downstream STAT1/4 phosphorylation culminating with T-bet gene transcription (5). Ultimately, IFNγ signaling initiates the Th1 differentiation program and IL-12 perpetuates it. In contrast, Th2 cells arise after occupancy of the IL-4Rα by its ligands IL-4 and IL-13, triggering JAK1/3 and subsequent activation of STAT6 (6), and leading to transcriptional regulation of the GATA3 target gene (5). More recently, the critical role of IL-17–producing Th cells (termed Th17 cells) in host defense against extracellular bacteria, maintenance of epithelium barrier integrity, and autoimmune pathogenesis has become increasingly clear. Within the immunologic microenvironment, IL-6 produced by activated dendritic cells (DCs) is a key factor in promoting Th17 differentiation via STAT3 and retinoic acid receptor–related orphan receptor γ (RORγt) induction (7) with IL-23 critical for memory Th17 *in vivo* function (3, 8).

## Atopic Dermatitis

Atopic dermatitis (AD) is a chronic, inflammatory skin disease that typically begins in early childhood and occurs more frequently in families with a history of other atopic diseases (bronchial asthma and/or allergic rhinoconjunctivitis). Overall, the prevalence of AD is up to 20% in children and 10% in adults, with rates varying geographically (9, 10). AD clinically manifests as recurrent eczematous lesions that negatively affect quality of life through sleep disturbances due to chronic itch (pruritus) (11, 12), increased likelihood of developing depression (13), and significant economic burden (14).

**Figure 3:**
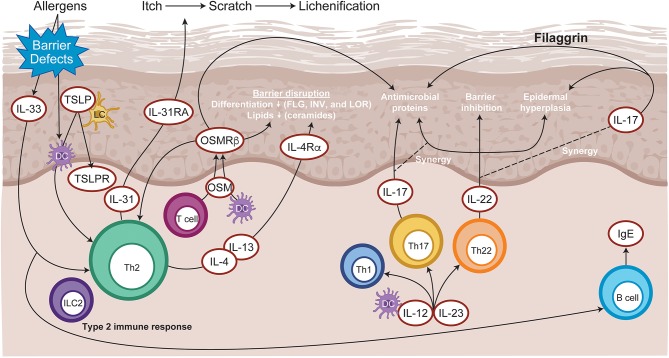
Immunopathogenesis of atopic dermatitis. Allergen entry through the disrupted **e**pidermal barrier stimulates keratinocytes to express cytokines, such as IL-33 and TSLP, which trigger ILC2 and Th2 cell mediated inflammation. Skin-resident dendritic cells take up exogenous and self-antigens released from damaged cells and promote type 2 immunity. CD8+ T cells infiltrate atopic dermatitis skin and activate Th2 cells to further release IL-4 and IL-13, which promotes IgE class switching. Cytokines released from skin infiltrating Th17 and Th22 lymphocytes synergize, leading to further barrier impairment and epidermal hyperplasia. DC, dendritic cell; FLG, Filaggrin; Ig, immunoglobulin; IL, interleukin; ILC2, type 2 innate lymphoid cells; INV, Involucrin; LC, Langerhans cell; LOR, Loricrin; OSM, Oncostatin M; OSMRβ, Oncostatin M receptor β; Th, T helper; TSLP, thymic stromal lymphopoietin.

The cellular infiltrate of AD lesions mainly consist of CD4^+^ T cells, which are considered key drivers of inflammation (15). Lesional skin is characterized by an overexpression of inflammatory Th2-cytokines (IL-4, IL-13, IL-31), and Th22-cytokines (IL-22) (16). Crucially, the cytokines IL-4, IL-13, IL-31, and IL-22 require JAK-STAT downstream signaling (3) for their biological function (Figure 3). Spontaneous and induced rodent dermatitis models have been extensively used to explore the effectiveness of small-molecule JAK inhibitors on reducing inflammation. Delgocitinib (pan-JAK) inhibited skin inflammation in hapten-induced chronic dermatitis in mice, as evidenced by reduced levels of inflammatory cytokines in the skin and IgE in serum (17). In addition, momelotinib (JAK1/JAK2) downregulated IL-4 expression, reduced the skin severity scores and reduced total serum IgE levels in the 2,4-dinitrochlorobenzene (DNCB)-induced AD mice (18). Similarly, tofacitinib (JAK1/3) and oclacitinib (JAK1) inhibited the production of proinflammatory Th2 cytokines, including IL-4, in the toluene-2,4-diisocyanate (TDI) dermatitis model (19). Moreover, tofacitinib demonstrated anti-inflammatory activity in the oxazolone-induced chronic allergic contact dermatitis model (20).

Interleukin-22 is elevated in AD lesions and is associated with epidermal thickening, skin barrier disruption, and increased expression of thymic stromal lymphopoietin (TSLP) and IL-33 cytokines (21). In addition, IL-22 potently induces the expression of gastrin-releasing peptide, a neuropeptide pruritogen, in dermal cells, dermal afferent fibers, and skin innervating ganglion neurons that positively correlate with the scratching behavior (22). The relevance of IL-22 in AD pathogenesis was emphasized by the observation of sustained clinical improvements in patients with moderate to severe AD receiving anti–IL-22 therapy (23). IL-22 binds its cognate receptor comprising a heterodimeric complex of IL-22RA1 and IL-10R2 subunits, leading to activation of JAK1 and TYK2 and phosphorylation of STAT3 (24).

Thymic stromal lymphopoietin and IL-31 cytokines also significantly contribute to triggering of inflammatory itch, under the control of IL-4, IL-13, and IL-33 (25). Crucially, pruritogenic cytokines IL-31 and TSLP use JAK1 and JAK2 downstream signaling (26, 27). Additionally, preclinical evidence has confirmed that pharmacologic inhibition of the JAK-STAT pathway is sufficient for the amelioration of pruritus-associated dermatitis. Examples include oclacitinib, which is licensed for pruritus associated with allergic dermatitis in dogs (28). Similarly, topical application of ruxolitinib (JAK1/JAK2) ameliorated TSLP-induced inflammation in mice (29). In the TDI-induced mouse model of dermatitis, oclacitinib and tofacitinib inhibited itch symptoms and significantly reduced IL-31, tumor necrosis factor–α (TNFα), and TSLP cytokine secretions (19). Moreover, TSLP can activate tissue resident dendritic cells that promote the transformation of Th-naïve lymphocytes to the Th2 phenotype thereby facilitating tissue inflammation (30). Finally, neuronal IL-4Rα acting via JAK1 signaling can also significantly contribute to chronic itch (31).

Skin barrier disruption and the resulting continuous exposure to allergens are presumed to be responsible for the development of atopic dermatitis (AD). JAK1-mediated Th2 cytokines IL-4 and IL-13 acting in a STAT-dependent manner (32) negatively affect skin barrier integrity by inhibiting the expression of filaggrin, loricrin, and involucrin, resulting in destabilization of tight junctions (33, 34). JAK inhibition restored filaggrin and loricrin expression following *in vitro* pretreatment with IL-4 /IL-13 cytokines of human keratinocyte. Moreover, mice harboring a point mutation leading to JAK1-specific hyperactivation develop spontaneous skin barrier disruption and a dermatitis phenotype (35). Topical application of delgocitinib ameliorated spontaneous AD-like skin inflammation and barrier disruption in an NC/Nga “dry skin” mouse model and restored filaggrin levels in an experimental human skin graft model leading to improved barrier function (36). Moreover, downstream signaling of IL-4 and IL-13 also suppresses the induction of innate immune response genes, such as β-defensins (33), thereby facilitating skin microbiome dysbiosis, including aberrant *Staphylococcus aureus* colonization (37).

The role and activation of Th1 and Th17 cell-mediated responses require further elucidation, but these pathways appear to be overexpressed in chronic disease stages, children, and people of Asian ethnicity (38, 39).

Targeting the JAK family of kinases in AD has proven, in recent years, to be therapeutically beneficial. Oral tofacitinib administration was evaluated in 6 patients with moderate to severe AD and showed a promising reduction in skin severity (40). The next generation of orally administered JAKinibs includes baricitinib (JAK1/2) along with two JAK1 selective molecules, upadacitinib (JAK1) and abrocitinib (JAK1). In clinical trials in moderate to severe AD patients, oral administration of these JAKinibs significantly reduced the eczema area severity index (EASI) scores by more than 50%. More specifically, in one clinical trial (ClinicalTrials.gov identifier, NCT02925117), oral administration of upadacitinib (JAK1) resulted in 90% improvement in the eczema area severity index (EASI) score for ~50% of enrolled participants after 16 weeks of treatment (41). Results from two baricitinib phase 3 studies showed that more patients achieved an investigator global assessment (IGA) 0/1 with barcitinib 4 mg once daily (QD) and 2 mg QD than with placebo (42). Significant improvements in EASI and patient-reported outcomes were observed as early as Week 1 (Table 2) (53). In a recent phase 1b study (NCT03139981), ASN002, a pan JAK inhibitor that also inhibits spleen tyrosine kinase (SYK), showed a 50% improvement in EASI in 100% of participants within 4 weeks (56).

**Table 2:** Summary of JAK inhibitor use in the treatment of dermatologic conditions.

**Study drug**	**Target**	**Company**	**Dermatology indications**	**ClinicalTrials.gov identifier**	**Studystatus**	**References**
**ORAL JAK INHIBITORS**						
Tofacitinib	JAK 1/2/3	Pfizer	AD	NCT02001181 (Ph2)	Completed	(43)
			Psoriasis	NCT01710046 (Ph2) NCT01815424 (Ph3) NCT01309737 (Ph3) NCT01276639 (Ph3) NCT01519089 (Ph3) NCT01241591(Ph3) NCT01186744 (Ph3) NCT01163253 (Ph3)	Completed Completed Completed Completed Completed Completed Completed Completed	(44) (45) (46) (47) (48) (49)
			AA, alopecia totalis, alopecia universalis,	NCT02197455 (Ph2)	Completed	(50)
			Dermatomyositis	NCT03002649 (Ph1)	Ongoing	
			Discoid lupus erythematosus,	NCT03159936 (Ph1)	Ongoing	
			cutaneous lupus	NCT03288324 (Ph1/2)	Ongoing	
Abrocitinib (PF04965842)	JAK1	Pfizer	Psoriasis	NCT02201524 (Ph2)	Terminated	(51)
			AD	NCT02780167 (Ph2) NCT03915496 (Ph2) (JADE MOA) NCT03627767 (Ph2) NCT03349060 (Ph3) (JADE Mono-1) NCT03575871 (Ph3) (JADE Mono-2) NCT03422822 (Ph3) (JADE EXTEND) NCT03720470 (Ph3) (JADE Compare) NCT03796676 (Ph3) (JADE TEEN)	Completed Ongoing Ongoing Completed Completed Ongoing Ongoing Ongoing	
PF-06651600	JAK3	Pfizer	AA	NCT02974868 (Ph2) NCT03732807 (Ph2/3) (ALLEGRO-2b/3)	Completed Ongoing	
			Vitiligo	NCT03715829 (Ph2)	Ongoing	
PF-06700841	JAK1/ TYK2	Pfizer	Psoriasis	NCT02969018 (Ph2)	Completed	
			AA	NCT02974868 (Ph2)	Completed	
			Vitiligo	NCT03715829 (Ph2)	Ongoing	
PF-06826647	TYK2	Pfizer	Psoriasis	NCT03210961 (Ph1) NCT03895372 (Ph2)	Completed Ongoing	
Baricitinib	JAK1/2	Eli Lilly/ Incyte	Psoriasis	NCT01490632 (Ph2)	Completed	(52)
			AA	NCT03570749 (Phase 2/3)(BRAVE-AA1) NCT03899259 (Ph3) (BRAVE-AA2)	Ongoing Ongoing	
			AD	NCT02576938 (Ph2) NCT03334396 (Ph3) (BREEZE-AD1) NCT03334422 (Ph3) (BREEZE-AD2) NCT03334435 (Ph3) (BREEZE-AD3) NCT03428100 (Ph3) (BREEZE-AD4) NCT03435081 (Ph3) (BREEZE-AD5) NCT03559270 (Ph3) (BREEZE-AD6) NCT03733301 (Ph3) (BREEZE-AD7) NCT03952559 (Ph3) (BREEZE-AD-PEDS)	Completed Ongoing Completed Ongoing Ongoing Ongoing Ongoing Ongoing Ongoing	(53) (42) (42)
Ruxolitinib	JAK1/2	Incyte	AA	NCT01950780 (Ph2)	Completed	(54)
Itacitinib	JAK1	Incyte	Psoriasis	NCT01634087 (Ph2)	Completed	(55)
INCB054707	JAK1	Incyte	Hidradenitis suppurativa	NCT03569371 (Ph2) NCT03607487 (Ph2)	Completed Ongoing	
Upadacitinib	JAK1	AbbVie	AD	NCT03646604 (Ph1) NCT02925117 (Ph2) NCT03569293 (Ph3) (Measure Up 1) NCT03568318 (Ph3) (AD Up) NCT03738397 (Ph3) (Heads Up) NCT03607422 (Ph3) NCT03661138 (Ph3)	Ongoing Completed Ongoing Ongoing Ongoing Ongoing Ongoing	(41)
ATI-501	JAK1/3	Aclaris	AA, alopecia totalis, alopecia universalis	NCT03594227 (Ph2)	Completed	
**ORAL JAK INHIBITORS**						
ASN002	JAK1/2/3 Tyk2 SYK	Asana BioSciences	AD	NCT03139981 (Ph1) NCT03654755 (Ph2) NCT03531957 (Ph2) (RADIANT)	Completed Ongoing Ongoing	(56)
			Chronic hand eczema	NCT03728504 (Ph2)	Ongoing	
Filgotinib	JAK1	Galapagos NV	Cutaneous lupus	NCT03134222 (Ph2)	Ongoing	
GSK2586184	JAK1	GSK	Psoriasis	NCT01782664 (Ph2)	Completed	
BMS-986165	TYK2	BMS	Psoriasis	NCT03004768 (Ph1) NCT02931838 (Ph2) NCT03924427 (Ph3) NCT03624127 (Ph3) (POETYK-PSO-1) NCT03611751 (Ph3) (POETYK-PSO-2)	Completed Completed Ongoing Ongoing Ongoing	(57)
Lestaurtinib	JAK2	Teva/Cephalon	Psoriasis	NCT00236119 (Ph2)	Completed	
Peficitinib	JAK3	Astellas	Psoriasis	NCT01096862 (Ph2)	Completed	(58)
CTP-543	JAK1/2	Concert Pharma-ceuticals	AA	NCT03137381 (Ph2) NCT03898479 (Ph2) NCT03941548 (Ph2) NCT03811912 (Ph2)	Completed Ongoing Ongoing Ongoing	
**Study drug**	**Target**	**Manufacture**	**Dermatology indications**	**ClinicalTrials.gov** **identifier**		
**TOPICAL JAK INHIBITORS**						
Tofacitinib	JAK1/2/3	Pfizer	Psoriasis	NCT01831466 (Ph2) NCT01246583 (Ph2)	Completed Completed	(59) (60)
			AD	NCT02001181 (Ph2)	Completed	(43)
			AA	NCT02812342 (Ph2)	Completed	(61)
PF-06700841	JAK1/ TYK2	Pfizer	Psoriasis	NCT03850483 (Ph2)	Ongoing	
			AD	NCT03903822 (Ph2)	Ongoing	
Ruxolitinib	JAK1/2	Incyte	Psoriasis	NCT00820950 (Ph2) NCT00778700 (Ph2) NCT00617994 (Ph2)	Completed Completed Completed	(62) (63)
			AD	NCT03257644 (Ph2) NCT03011892 (Ph2) NCT03745638 (Ph3) (TRuE-AD1) NCT03745651 (Ph3) (TRuE AD2)	Ongoing Completed Ongoing Ongoing	(64, 65)
			AA	NCT02553330 (Ph2)	Terminated	
			Vitiligo	NCT03099304 (Ph2) NCT02809976 (Ph2)	Ongoing Completed	(66, 67)
ATI-502 (ATI-50002)	JAK1/3	Aclaris	AD	NCT03585296 (Ph2)	Completed	
			AA, alopecia totalis, alopecia universalis	NCT03315689 (Ph2-AU and AT) NCT03551821 (Ph2-eyebrow) NCT03354637 (Ph2) NCT03759340 (Ph2)	Completed Completed Ongoing Ongoing	
			Androgenetic alopecia	NCT03495817 (Ph2)	Ongoing	
			Vitiligo	NCT03468855 (Ph2)	Ongoing	
Delgocitinib (JTE-052)	JAK1/2/3 Tyk Syk	Japan Tobacco Inc.; Leo	AD	NCT03826901 (Ph1) NCT03725722 (Ph2) (mild to severe AD)	Ongoing Ongoing	
			Chronic hand eczema	NCT03683719 (Ph2)	Ongoing	
			AA	NCT02561585 (Ph2) NCT03325296 (Ph2-eyebrow)	Completed Terminated	
			Discoid lupus	NCT03958955 (Ph2)	Ongoing	

*AA, alopecia areata; AD, atopic dermatitis; JAK, Janus kinase; TYK, tyrosine kinase*.

Topical administration of tofacitinib to patients with mild to moderate AD in a clinical trial (NCT02001181) demonstrated significant improvement in EASI scores at Week 4 (43) and improvement in pruritus as early as Day 2. In a phase 2 study (NCT03011892) involving patients with mild to moderate AD receiving ruxolitinib cream, mean percentage change from baseline at Week 4 in EASI score demonstrated a significant improvement and was non-inferior to triamcinolone. Interestingly, significant reductions in itch were noted as early as 1 day after initiation of therapy (64, 65). More recently, pilot studies of topical ATI-502 (JAK1/3) solution (NCT03585296) and PF-06700841 (JAK1/TYK2) cream (NCT03903822) in AD are ongoing. Given the early successes of JAKinibs in AD, ongoing investigation and evaluation is expected to further elucidate the differential effects of JAK selectivity.

## Alopecia Areata

Alopecia areata (AA) is an autoimmune disease resulting in partial or complete nonscarring hair loss, with a prevalence of ~1.7 to 2.1% (68). Susceptibility to AA is indiscriminate between the sexes and ethnicities, with initial disease onset often occurring before the third decade of life. Early symptoms are typically characterized by small, well-defined patches of hair loss that may spontaneously resolve with time; however subsequent relapses occur in around a third of cases. Spontaneous remission is rare in patients with alopecia totalis or alopecia universalis. To date, no FDA- or European Medicines Agency–approved treatments exist.

Multiple lines of evidence have demonstrated that AA pathogenesis is autoimmune in nature, with loss of immune privilege and associated T cells infiltration selectively attacking growth at the hair follicle (i.e., anagen phase) (69–72). Healthy hair follicles achieve immune privilege at the anagen phase by downregulation of expression of major histocompatibility complex (MHC) class I and class II molecules (70, 73) and by expression of NK and CD8^+^ cell inhibitors, such as macrophage migration inhibitory factor (MIF) and transforming growth factors (TGF) β1 and β2, which generate an immunosuppressive microenvironment (74–76). Importantly, hair follicle epithelial stem cells are usually spared during the autoimmune attack, which provides a potential mechanism of hair growth recovery with effective anti-inflammatory treatment (70).

Many different mammalian species, including rodents, are susceptible to AA and this has facilitated preclinical models for the elucidation of cellular and molecular immune pathways (77, 78). The inbred C3H/HeJ strain spontaneously develops alopecia in up to 20% of mice via an IFNγ- and inflammasome-sensitive mechanism (79); however recipient C3H/HeJ animals that receive skin grafts from donor alopecic C3H/HeJ mice develop an accelerated phenotype with nearly 100% disease penetrance (80). Transfer or deletion of effector CD8^+^ T cells is sufficient to induce or block disease in preclinical models (70, 71, 81), which in consistent with the observation that cytotoxic CD8^+^NKG2D^+^ T cells expressing granzyme B (82) infiltrate around the hair follicles and are major contributors of hair loss (81, 83).

Global transcriptional analyses of mouse and human affected skin identified expression signatures indicative of cytotoxic T-cell infiltration, such as increased production of IFNγ and γ-chain (γc) cytokines, including IL-15 (81, 84). Furthermore, inhibiting IFNγ either by genetic deletion or neutralizing antibody significantly ameliorates AA development and severity (85), supporting the hypothesis that IFNγ drives AA pathogenesis by inducing ectopic expression of MHC molecules and ligands that stimulate NK-cell receptors (NKG2D) in the anagen hair bulb leading to the collapse of the hair follicle immune privilege (70, 86–88). An important cellular source of IFN is plasmacytoid dendritic cells (pDCs), which are normally absent from healthy skin, but migrate into tissues in response to inflammatory stimuli or infection. Infiltrating pDCs have been identified around hair follicles of patients with AA (89) and, upon activation, produce large quantities of type I IFNs (90).

**Figure 4:**
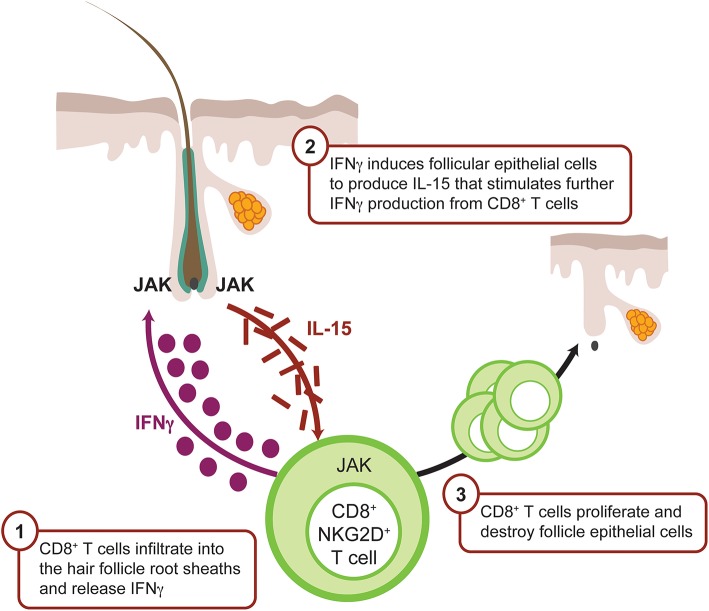
IFNγ-driven inflammation in alopecia areata is JAK mediated. CD8^+^ T cells infiltrate the dermis, localize to the hair follicle bulb, and release IFNγ. IFNγ binds the IFN receptor on the surface of the follicular epithelial cell, which in turn signals via JAK1 and JAK2 to promote production of IL-15, a mediator of CD8^+^ T-cell activation. IL-15 binds IL-15 receptor on the CD8^+^ T cell surface, resulting in signaling via JAK1 and JAK3 to enhance the production of IFNγ and amplify the feedback loop. CD8^+^ T cells then attack the hair follicle, which causes hair loss. CXCL, chemokine (C-X-C motif) ligand; IFN, interferon; JAK, Janus kinase.

The IFNγ-induced chemokine receptor CXCR3 and its ligands CXCL9 and CXCL10 are upregulated around hair follicles during early AA pathogenesis (Figure 4), thereby facilitating lymphocyte recruitment (82, 91). CXCR3 is primarily expressed on Th1 CD4^+^ T cells, CD8^+^ T cells, NK, and PDCs during skin inflammation (92), whereas CXCR3 ligands are secreted by many tissue resident cells, including dendritic cells.

Like IFNγ, IL-15 enhances innate and self-reactive memory T-cell immunity, including autoimmune disease pathogenesis (84, 93), and signals via the JAK1/3 pathway with downstream STAT-5 activation (94). Similarly blocking IL-2 or IL-15 receptor beta (IL-15Rβ) ameliorated disease development by inhibiting CD8^+^NKG2D^+^ T-cell accumulation in the skin (81).

The combination of published genome-wide association studies in patients with AA that highlighted JAK signaling (87, 95) and the knowledge that IFNγ primarily signals through JAK1/2 and IL-15 mostly through JAK1/3, provided a compelling rationale for the exploration of small-molecule JAK inhibitors in AA disease (81, 96). Preclinical evaluation of orally administered ruxolitinib and tofacitinib in the skin graft C3H/HeJ mouse model demonstrated disease prevention (81). Marked decreases of CD4, CD8, and MHC class I and II as well as a reduced numbers of CD8^+^/NKG2D^+^ cells were observed in the skin (81). Subsequently, prophylactic and therapeutic baricitinib treatment ameliorated disease and normalized the Alopecia Areata Disease Activity Index (ALADIN) IFNγ gene expression signature (80). Topical administration of JAK inhibitors reversed AA in C3H/HeJ mice (81); however, murine skin is significantly thinner and easier to penetrate, and, therefore the translational validity of these data is still unknown. In addition to its proinflammatory activity, IFNγ-induced JAK/STAT signaling and the recruitment of CD8^+^ T cells through CXCL9 and CXCL10 can directly interfere with the hair growth cycle via suppressed proliferation and activation of hair stem cells (97) and reduction of angiogenesis (98).

Several case studies have reported improvement of AA in patients who received JAK inhibitors for other autoimmune/autoinflammatory disorders or JAK-STAT gain-of-function mutation diseases (99–103).

Oral tofacitinib has been tested in two open-label studies (NCT02197455 and NCT02312882) and several case reports. In one trial, tofacitinib 5 mg twice daily (BID) was given to patients with severe AA, alopecia totalis, or alopecia universalis. After the 12-week treatment period, nearly two-thirds of patients showed some hair regrowth and 32% of patients achieved a 50% improvement in their Severity of Alopecia Tool (SALT) score (50). The second smaller open-label study in moderate to severe AA demonstrated improved results by increasing the dose of tofacitinib to 10 mg BID (104). Recently, two retrospective studies showed successful treatment of severe AA, alopecia totalis, or alopecia universalis for up to 18 months using tofacitinib, with 58% achieving a 50% improvement 20% achieving a 90% improvement in their SALT score (105–107). Oral ruxolitinib was tested in an open-label study in 12 patients with moderate to severe AA and treatment with 20 mg ruxolitinib BID for 6 months was associated with ≥50% improvement in SALT score for 75% of patients (NCT01950780) (54). Regrowth was seen, in patches as soon as 1 month after study medication was initiated. Following cessation of treatment, shedding was observed, suggesting that pharmacologic JAK inhibition suppresses AA pathogenesis but does deplete autoreactive lymphocytes. Topical formulations of ruxolitinib, tofacitinib, ATI-502 (JAK1/3), and delgocitinib have reported mixed efficacy results in case studies and small proof-of-concept clinical trials (61, 108, 109). At present, there are several clinical trials testing topical JAK inhibitors in patients with different forms of AA, but published results are not yet available (Table 2).

## Psoriasis

Psoriasis is a chronic, autoimmune, erythematosquamous dermatosis disorder that affects 2 to 3% of the world population. Skin lesions appear with scaling and redness (110) and are characterized by excessive keratinocyte proliferation (acanthosis), as well as retention of nuclei in the stratum corneum owing to aberrant keratinocyte differentiation (parakeratosis). Multiple inflammatory cell populations are observed within the lesions, including T cells, B cells, neutrophils, and DCs (110). Infiltrating autoreactive T lymphocytes, mainly represented by Th17, Th1, and Th22 cells, release IL-17, IFNγ, IL-22, and TNFα to potentiate disease pathogenesis. All of these cytokines induce keratinocyte-mediated recruitment and activation of additional DCs and lymphocytes, thereby perpetuating the pathogenic cycle (111, 112).

Many of the critical pathogenic mediators of psoriasis are linked to the JAK-STAT signaling pathway. For example, IL-23 engagement with its cognate receptor uses JAK1/2/TYK2 signaling, resulting in downstream STAT3 and STAT4 activation. Within psoriatic skin, dendritic cells and macrophages produce IL-23, which promotes Th17 cell expansion and survival (113). Furthermore, IL-23 together with IL-1β activates γδ T cells to amplify IL-17 production (114). Th17 and γδ T cells found in psoriatic skin are the primary source of IL-22, and this cytokine triggers reduced differentiation, increased proliferation, and acanthosis in psoriatic keratinocytes via STAT3 activation (115). IL-22 binds to its IL-10R2 and IL-22R1 heterodimeric cell surface receptor coupled to JAK1/TYK2 and STAT3 signaling (111, 116). Moreover, gene polymorphisms of *IL23A, IL23R, STAT3, RUNX3*, and *TYK2* have also been identified as susceptibility factors for developing psoriasis (117). More recently, JAK1 expression has been reported to positively correlate with disease duration and Psoriasis Area and Severity Index (PASI) (118) score. Within the inflamed tissue psoriatic lesion microenvironment, other cytokines, such as IL-6 and IL-21, can enhance IL-17 production from Th17 cells in a JAK-STAT–dependent manner (119, 120).

Various rodent models have mechanistically evaluated the importance of JAK-STAT signaling in psoriasis-like lesion formation and disrupted barrier function. Intradermal injection of IL-23 induces a psoriasis-like pathophysiology in mice (121). Oral administration of delgocitinib or topical administration of ruxolitinib significantly inhibited ear swelling (29, 122), and efficacy was associated with reduced IL-22 expression (29). Tofacitinib, modulates both innate and adaptive immunity leading to inhibited pathogenic Th17 cell differentiation via reduced IL-23 expression (123). In human keratinocyte cultures activated with psoriasis-relevant proinflammatory cytokines, tofacitinib suppressed expression of IFNγ-dependent inflammatory genes and normalized keratinocyte responses. Similarly, in the imiquimod-induced psoriasis model that is IL-23/IL-17/IL-22–dependent (121), tofacitinib significantly reduced epidermal thickening and IL-17^+^ or IL-22^+^ lymphocyte infiltration into the dermis (124). Furthermore, a small molecule JAK3 / SYK inhibitor (R348, Rigel Pharmaceuticals), attenuated T-cell–dependent psoriasiform skin lesions in the CD18 mutant PL/J mouse model, including significant reductions in epidermal and dermal lesion scores (125). In T cells, IL-12 induces IFNγ production, IL-23 enhances the differentiation of Th17 cells, and both require TYK2 signaling (126, 127). TYK2 knockout reduced inflammatory response and limited epidermal hyperplasia in the intradermal IL-23 model (128). Furthermore, TYK2-deficient mice were more resistant to several Th1 and Th17 cells autoimmune disorders, including imiquimod-induced psoriasis-like dermatitis (128). The combined JAK1/TYK2 inhibitor, SAR-20347, demonstrated *in vitro* and *in vivo* concentration-dependent reduction of IL-12, IL-22, and IFNγ-mediated inflammation and tissue pathology in the imiquimod-induced psoriasis model (129). Finally, experimentally induced skin trauma in the keratin5.Stat3C transgenic mice (130), which constitutively overexpresses active STAT3 in keratinocytes, develops T-cell– and IL-23–dependent psoriasis-like lesions. Topical administration of a STAT3 inhibitor prevented disease symptoms (130–132). These preclinical findings are consistent with the postulate that the JAK-STAT pathway plays a central role in psoriasis pathogenesis.

Clinically, the efficacy of oral tofacitinib in moderate to severe plaque psoriasis was demonstrated in two phase 3 randomized controlled trials (46). Tofacitinib at 10 mg BID was determined to be non-inferior to etanercept (50 mg subcutaneously twice weekly) (47). Baricitinib was reported to be efficacious in moderate to severe psoriasis in a phase 2 trial (NCT01490632). In this 12-week dose-ranging study, a 75% reduction in PASI was achieved by 43% and 54% of patients treated with baricitinib 8 and 10 mg QD, respectively (52). Itacitinib (JAK1) was evaluated in a phase 2 dose-escalation study in which patients experienced a significant improvement in the Physician Global Assessment (PGA) score at Week 4 with itacitinib 600 mg QD vs. placebo (NCT01634087) (55). Peficitinib (JAK1/3) reported improvements in PASI, PGA, and body surface area at higher dose (50 mg QD) at Day 42 in a phase 2 study (58). In another phase 2 study, 57% of patients treated with GSK2586184 (JAK1) 400 mg QD achieved a 75% reduction in PASI at Week 12 (133).

Topical tofacitinib has been tested in patients with psoriasis, with conflicting results (52, 60, 134). Three psoriasis clinical trials have been completed using topical ruxolitinib cream. In a phase 2 vehicle-controlled study in mild to moderate psoriasis (NCT00778700), ruxolitinib reported PASI reduction, although no clear dose-response was observed. A subsequent trial in 29 patients with psoriasis compared ruxolitinib cream to two active comparators (calcipotriene 0.005% cream and betamethasone dipropionate 0.05% cream; NCT00820950). Both ruxolitinib 1% QD and 1.5% BID achieved clinical efficacy, with 1.5% BID topical ruxolitinib cream being non-inferior to active comparators (62). Finally, a third study conducted in 25 patients with limited psoriasis (covering <20% of the body surface area; NCT00617994) showed that epidermal hyperplasia and dermal inflammation were reduced with ruxolitinib in most patients, along with immunohistochemical markers of inflammation (CD3, CD11c, Ki67, and K16). No significant inhibition of phosphorylated STAT3 in peripheral blood cells was observed, suggesting limited systemic exposure (63). A number of other JAK inhibitors have been studied in psoriasis (Table 2).

## Vitiligo

Vitiligo is a chronic, autoimmune depigmenting disorder that results from destruction of melanocytes, causing white spots on the affected skin. The global vitiligo prevalence is ~0.5 to 2.0% and varies geographically, with no epidemiologic differences between sexes or races (135, 136). Vitiligo can be stigmatized by society, resulting in a significant impact to patient quality of life (137, 138). It is therefore inappropriate to categorize vitiligo as simply a cosmetic problem.

**Figure 5:**
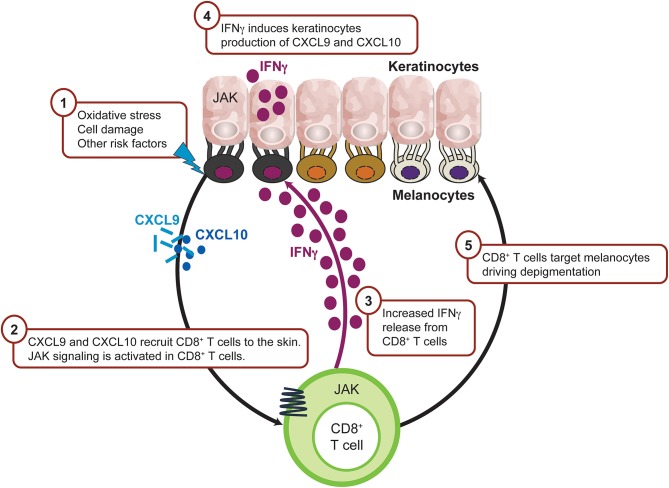
IFNγ-driven inflammation in vitiligo is JAK mediated. Intrinsic and/or extrinsic factors induce the cellular stress response in melanocytes, which then activates innate immunity within the skin to trigger the initial inflammation that leads to autoimmunity. As a result, CXCL9 and CXCL10 are released from keratinocytes leading to recruitment of CD8^+^ T cells. Activated CD8^+^T cells produce IFNγ which trigger more CXCL9 and CXCL10 production from keratinocyte through JAK1 and JAK2 signaling and recruit more CD8^+^ T cells to the inflamed sites. CD8^+^ T cells then destruct melanocytes and lead to depigmentation. CXCL, chemokine (C-X-C motif) ligand; IFN, interferon; IL, interleukin; JAK, Janus kinase; NKD2D, natural killer group 2D.

In vitiligo, the frequency of anti-melanocyte CD8^+^ T cells in the blood and skin correlates with disease severity, and lesional CD8^+^ T cells *in vitro* induce melanocyte apoptosis in unaffected skin (139, 140). These data support the rationale that cytotoxic T lymphocytes are directly responsible for melanocyte destruction in human vitiligo (Figure 5). Expression analysis reveals an IFNγ-specific signature that is associated with infiltrating autoreactive CD8^+^ T cells (140, 141). Transcriptome analysis on the skin and blood of patients with vitiligo revealed IFNγ-induced chemokines CXCL10 and CXCL9 were increased (142, 143), which is consistent with the observed abundance of autoreactive T cells expressing the cognate CXCR3 receptor (144). Furthermore, serum CXCL10 levels were associated with Vitiligo Area Scoring Index (VASI) of patients with progressive vitiligo, suggesting that the CXCL10/CXCR3 axis mediates T-cell recruitment into the skin of progressive vitiligo.

Consistent with active human vitiligo reports, an adoptive transfer of melanocyte-specific CD8^+^ mouse model shows epidermal depigmentation but sparing of the hair follicle. Mechanistic studies, including neutralizing antibodies, have demonstrated that depigmentation is IFNγ-dependent via the local accumulation of melanocyte-specific CD8^+^ T cells within the skin. Adoptive transfer of CXCR3-deficient T cells or inhibition of CXCL10 signaling ameliorated overall disease phenotype, whereas CXCL9 promoted autoreactive T-cell recruitment to the skin but did not significantly contribute to effector function (141, 145). Keratinocytes appear to significantly contribute to the disease process as major chemokine producers via IFN signaling, resulting in augmented autoreactive T-cell homing to the epidermis (146).

Given the apparent critical role for IFNγ in driving vitiligo inflammation and its downstream signaling dependent on the JAK1-JAK2 heterodimer, it is perhaps not surprising that intense and diffuse JAK1 expression is more present within vitiliginous skin compared with healthy tissue. Moreover, high JAK1 expression was associated with short disease duration and a lower percentage of surviving melanocytes (118, 147, 148).

Multiple case reports suggested that orally administered JAK inhibition significantly modulated the vitiligo autoimmune response and facilitated repigmentation (102, 108, 149). Another possible approach to diminish local inflammation and promote repigmentation in vitiligo, but minimize systemic drug exposures, is the use of topical JAK inhibitors. Recently, Rothstein et al. reported a very small open-label trial without placebo control (NCT02809976) in which nine patients completed the 20 week study period. Twice daily ruxolitinib cream demonstrated time-dependent improvement in facial VASI (F-VASI) in the majority of the enrolled vitiligo patients (67). Recently, ruxolitinib cream was tested in a randomized, double-blinded, dose-ranging, vehicle-controlled, phase 2 study in 157 adult patients with vitiligo (NCT03099304). The results show that significantly more patients treated with ruxolitinib cream for 24 weeks achieved a ≥50% percent improvement from baseline in the facial VASI score compared with patients treated with a control vehicle [(66); World Congress of Dermatology; June 2019; Milan, Italy].

## Conclusions

Despite phenotypic differences in the inflammatory mediators responsible for driving disease pathogenesis, these aforementioned dermatoses are characterized by increased inflammatory mediators that signal through the JAK-STAT pathway.

JAK inhibitors are emerging as an exciting class of treatments in the field of dermatology. In murine models of skin inflammation, JAK inhibitors significantly modulated key mechanistic phenotypes that correspond with clinical readouts, such as acanthosis and pruritus. Early phase clinical reports confirmed the positive concept of JAK-STAT antagonism in dermatology, and randomized clinical trials have shown promising results in AD, psoriasis, and vitiligo. Encouraging data were observed in a proportion of AA participants; however, additional studies are needed to fully elucidate the disease pathophysiology and the role for JAK-STAT inhibition.

Larger clinical studies of oral and topical JAK inhibitors in AD, psoriasis, and vitiligo are currently ongoing. These pivotal trials are expected to provide additional insight into the efficacy and safety of JAK inhibitors in dermatology. Safety information for Jakinibs in inflammatory disease indications is mostly based on randomized clinical trials for investigational uses and extension studies. Recent, comprehensive, summaries of the key laboratory changes and clinical adverse events have been reported (150, 151).

Based on the promising results so far and the large number of ongoing clinical trials, it is possible that JAK inhibitors will become an important part of the dermatologist's treatment armamentarium in the future.

## Author Contributions

All authors listed have made a substantial, direct and intellectual contribution to the work, and approved it for publication.

### Conflict of Interest

MH, FK, and PS are employees of Incyte Corporation.

## Abbreviations

AA: Alopecia areata
ACT: Nuclear factor NF-kappa-B activator 1
AD: Atopic dermatitis
BID: Twice daily
CD: Cluster of differentiation
CXCL: Chemokine (C-X-C motif) ligand
CXCR: Chemokine (C-X-C motif) receptor
DC: Dendritic cell
DNCB: 2,4-dinitrochlorobenzene
EASI: Eczema Area Severity Index
EPO: Erythropoietin
FDA: US Food and Drug Administration
FLG: Filaggrin
GATA-3: GATA transcription factor 3
GH: Growth hormone
GM-CSF: Granulocyte-macrophage colony-stimulating factor
IFN: Interferon
Ig: Immunoglobulin
IL: Interleukin
ILC2: Type 2 innate lymphoid cells
IGA: Investigator global assessment
INV: Involucrin
JAK: Janus kinase
JAKinibs: Janus kinase inhibitors
LOR: Loricrin
MHC: Major histocompatibility complex
NK: Natural killer cell
NKD2D: Natural Killer Group 2D
NKD2DL3: Natural Killer Group 2D ligand 3
PASI: Psoriasis Area and Severity Index
pDC: Plasmacytoid dendritic cell
PGA: Physician Global Assessment
QD: Once daily
OSM: Oncostatin M
OSMRβ: Oncostatin M receptor β
PI3K: Phosphoinositide 3-kinases
RAET1L: Retinoic acid early transcript 1L
RORγt: Retinoic acid receptor-related orphan receptor γ
SALT: Severity of Alopecia Tool
STAT: Signal transducer and activator of transcription
T-bet: T-box transcription factor TBX21
TGF: Transforming growth factor
Th: T helper
TNFα: Tumor necrosis factor alpha
TPO: Thrombopoietin
TSLP: Thymic stromal lymphopoietin
TYK2: Tyrosine kinase 2
VASI: Vitiligo Area Scoring Index.
